# PBPK‐Led Assessment of Antimalarial Drug Concentrations in Breastmilk: A Strategy for Optimal Use of Prediction Methods to Guide Decision Making in an Understudied Population

**DOI:** 10.1002/psp4.13311

**Published:** 2025-02-11

**Authors:** Lisa M. Almond, Khaled Abduljalil, Amita Pansari, Beata Kusmider, Hannah M. Jones, Karen Rowland Yeo, Iain Gardner, Muhammad Faisal, Anne Claire Marrast, Myriam El Gaaloul, Jörg J. Möhrle, Nada Abla

**Affiliations:** ^1^ Certara Predictive Technologies, Simcyp Division Sheffield UK; ^2^ MMV Medicines for Malaria Venture Geneva Switzerland

**Keywords:** lactation, malaria, physiologically based pharmacokinetics

## Abstract

Treatment of breastfeeding mothers with malaria is challenging due to the lack of information describing drug exposure in milk and the daily dose to the breastfed infant. Physiologically based pharmacokinetic (PBPK) modeling was used to predict milk‐to‐plasma (M/P) ratios, infant daily doses (IDD) and relative infant doses (RID) for five antimalarials with clinical lactation data (chloroquine, pyrimethamine, piperaquine, mefloquine and primaquine). In all cases, RID was correctly categorized as above or below the WHO proposed cut‐off of 10% using two prediction models. Predicted M/P ratios were within 2‐fold of observations for 63% of studies using both models (75% and 100% were within 3‐fold for Models 1 and 2, respectively). M/P ratios, IDD and RID were predicted prospectively for seven antimalarials. RID was < 10% for amodiaquine, dihydroartemisinin, proguanil, and pyronaridine, and > 10% for lumefantrine and tafenoquine. For atovaquone, RID was > 10% with Model 1 but not Model 2. Predicted IDD were considerably lower than licensed doses for infants except for lumefantrine (Model 2) and tafenoquine (not licensed in < 2 years). Predictions were sensitive to drug properties (plasma protein binding and lipophilicity) and milk properties (creamatocrit and pH). This analysis demonstrates the utility of PBPK to predict milk exposure in the absence of clinical lactation information. These prediction methodologies can be used, alongside any licensed dosing information for < 1 year‐olds, to evaluate whether a clinical lactation study is necessary and to inform drug label or policy recommendations. The ultimate goal is to better inform optimal treatment for lactating women supporting malaria eradication.

**Summary**

- What is the current knowledge on the topic?
  ○ Access to malaria treatment for lactating women is crucial. Despite that, there is little pharmacokinetic data on transfer of antimalarials to breastmilk and the daily dose a breastfed infant receives.
- What question did this study address?
  ○ How can physiologically based pharmacokinetic (PBPK) modeling be used to predict milk exposure and infant dose of antimalarials to support decision making?
- What does this study add to our knowledge?
  ○ We developed a PBPK‐based strategy to predict the transfer of antimalarials to breastmilk and calculate the infant daily doses. We verified the modeling approach with available clinical lactation data for antimalarials and then used it to predict milk exposure for seven drugs without clinical lactation data. We identified key drug and milk properties that the predictions are particularly sensitive to and provide recommendations on when additional characterization is required.
- How might this change drug discovery, development, and/or therapeutics?
  ○ This work proposes a strategy that can be used to predict breastmilk exposure and infant dose to guide the necessity and design of clinical lactation studies and to better inform label or policy recommendations.

## 1 Introduction

Malaria is still a highly prevalent life‐threatening transmissible disease. The WHO reported around 249 million estimated global cases and 608,000 deaths in 2022 [1]. Interventions including the use of antimalarial drugs have helped to reduce mortality rates.

The WHO and UNICEF recommend that infants commence breastfeeding within the first hour of birth and be exclusively breastfed for the first 6 months of life. From the age of 6 months, they should begin eating safe and adequate complementary foods while continuing to breastfeed for up to 2 years of age or beyond [2, 3] and in malaria endemic regions, this is the norm [4]. During this period, maternal immunity undergoes a slow return to normal after the immunosuppression related to pregnancy [5, 6, 7] and is associated with an increased rate of malarial infection [8] and subsequent mortality, especially in geographic regions with high HIV prevalence. Access to malaria treatment for lactating women is therefore crucial. Although the WHO recommend artemisinin‐based combination therapies to be administered to breastfeeding women [9], there is a scarcity of milk pharmacokinetic data even for first‐line therapies [4], and limited information provided in drug labels. While recent regulatory guidance requires drug developers to consider diversity in clinical trial design [10], breastfeeding mothers and their breastfed infants remain an understudied group, partly due to the frequent exclusion of breastfeeding mothers from clinical studies.

To improve access to effective and informed malaria treatment postpartum, clinical lactation studies are needed to quantify drug concentrations in milk, the likely dose that a breastfed infant can receive through milk intake, and the extent to which the drug becomes available in the infant's systemic circulation. However, clinical lactation studies present challenges in terms of ethics, study design, recruitment, sampling, bioanalytics, guided breaks in breastfeeding (when feasible) and the interpretation of findings in the context of measured maternal variables, such as milk pH and creamatocrit [11]. Hence, it is not feasible to carry out clinical lactation studies for all existing antimalarials. Furthermore, animal studies can indicate the presence or absence of a drug in milk but cannot accurately predict human drug milk concentrations in milk [12, 13]. A practical approach using predictions of milk exposure and infant dose is, therefore, beneficial to evaluate whether a clinical evaluation is warranted. The ultimate goal is to update drug label or policy recommendations once the robustness of the models has been confirmed.

Physiologically based pharmacokinetic (PBPK) modeling combines the physiological characteristics in a population of interest with drug properties and therefore offers a mechanistic approach to predict time‐varying concentrations of drug in lactating mothers, their milk and their breastfed children [14, 15, 16].

In this work, published PBPK models for antimalarial drugs were extended to predict milk‐to‐plasma (M/P) ratios using drug physicochemical information (lipophilicity, the fraction unbound in plasma (fu), ionization) and physiological parameters such as milk pH, and creamatocrit [14, 15]. These were then used to predict the drug concentration in milk over time, the infant daily dose (IDD) and the relative infant daily dose (RID) for each compound. The simulated data were compared to clinical lactation data for five drugs where lactation studies had been conducted. Sensitivity analyses were performed to assess the impact of drug and milk properties on the predicted M/P ratio to determine the key parameters that need to be measured either in vitro or in clinical lactation studies.

## 2 Methods

### 2.1 Lactation Modeling

Two published lactation quantitative structure–activity relationship (QSAR) models, a phase‐distribution [17, 18] (Model 1) and a natural log‐transformed distribution model [18] (Model 2) were coupled with a maternal PBPK model [15], to predict the M/P ratios assuming a mature milk composition. The M/P ratios calculated using both methods were then used to predict milk exposure, the IDD and RID using the predicted average drug concentration in the milk for each simulated individual, assuming a constant milk intake of 150 mL/kg/day.

#### 2.1.1 Calculation of M/P Ratio

Model 1 incorporates physiological aspects of drug partitioning to the aqueous and lipid phases of milk, as well as the degree of free drug ionization in milk and maternal plasma to calculate the M/P ratio. This phase‐distribution model has been described in detail elsewhere [17, 18]:

(1) $$\begin{array}{c} \frac{M}{P}=\frac{\mathrm{fu}\cdot f_{p}^{\;\mathrm{un}}}{f_{\mathrm{mk}}^{\;\mathrm{un}}}\cdot \frac{1}{{\mathrm{fu}}_{\mathrm{mk}}\cdot \left(\frac{1}{1+f_{\mathrm{fat}}\cdot \left({\mathrm{fu}}_{\mathrm{mk}}\cdot P_{\text{milk}}-1\right)}\right)} \end{array}$$

where $\frac{1}{1+f_{\mathrm{fat}}\cdot \left({\mathrm{fu}}_{\mathrm{mk}}\cdot P_{\text{milk}}-1\right)}$ represented the skimmed to whole milk ratio (S/W).

The terms in this equation are defined below.

Model 2 uses the same input parameters (model components) as Model 1, but differentiates between acidic and basic drugs with respect to the weighting it gives to these components based on regression analysis [18]. This log (natural log, Ln)‐transformed distribution model takes the following form for basic (Equation 2) and acidic (Equation 3) drugs:

(2) $$\mathrm{Ln}\frac{M}{P}=0.025+2.28\ln\left(\frac{\mathrm{Mu}}{\mathrm{Pu}}\right)+0.89\;\ln\mathrm{fu}+0.51\;\ln K$$

(3) $$\mathrm{Ln}\frac{M}{P}=-0.405+9.36\ln\left(\frac{\mathrm{Mu}}{\mathrm{Pu}}\right)-0.69\;\ln\mathrm{fu}-1.54\;\ln K$$

where,

(4) $$K=\left(\frac{1-f_{\mathrm{fat}}}{\;{\mathrm{fu}}_{\mathrm{mk}}}\right)+f_{\mathrm{fat}}\cdot P_{\text{milk}}$$

Terms in the equations:

fu, Individualized unbound fraction of the drug in maternal plasma; $f_{\mathrm{mk}}^{\;\mathrm{un}}$, Unionized fraction of the drug in milk; $f_{p}^{\;\mathrm{un}}$, Unionized fraction of the drug in plasma; $f_{\mathrm{fat}}$, Individualized fractional volumes of the fat component in milk, also known as creamatocrit, sampled from a log normal distribution of values across the virtual population with a mean of 6.2 g/100 mL of milk (coefficient of variation (CV) = 33%); ${\mathrm{fu}}_{\mathrm{mk}}$, unbound fraction of the drug in skimmed milk for each virtual subject calculated using the following equation [19]:

(5) $${\mathrm{fu}}_{\mathrm{mk}}=\frac{{\mathrm{fu}}^{0.448}}{\;0.000694^{0.448}+{\mathrm{fu}}^{0.448}}$$

$P_{\text{milk}}$ is the milk lipid‐to‐ultrafiltrate partition coefficient. Unless otherwise stated the mature milk pH of 7.2 (${\mathrm{Log}D}_{7.2}$) was used and $P_{\text{milk}}$ was calculated according to the following equation [18]:

(6) $$P_{\text{milk}}=10^{\left(-0.88+1.29\;{\mathrm{Log}D}_{7.2}\right)}$$

${\mathrm{Log}D}_{7.2}$ was calculated from the drug octanol‐to‐water partitioning ratio (Log*P*_*o*:*w*_) to account for the ionization state at the reported milk pH where available or at the standard pH of 7.2 used for mature milk.

$\frac{\mathrm{Mu}}{\mathrm{Pu}}$ is the steady state distribution of unbound drug between milk and plasma obtained as follows:

$$\frac{\mathrm{Mu}}{\mathrm{Pu}}=\frac{f_{p}^{\;\mathrm{un}}}{f_{\mathrm{mk}}^{\;\mathrm{un}}}$$

where, unionized fraction of the drug in milk $f_{\mathrm{mk}}^{\;\mathrm{un}}$ and unionized fraction of the drug in plasma $f_{p}^{\;\mathrm{un}}$ are calculated using the compound *p*Ka(s), and the plasma and milk pH using the Henderson‐Hasselbalch equation:

For a monoprotic base:

(7) $$f_{p}^{\;\mathrm{un}}=\frac{1}{1+10^{\left(p\mathrm{Ka}–{\mathrm{pH}}_{\text{Plasma}}\right)}}$$

(8) $$f_{\mathrm{mk}}^{\;\mathrm{un}}=\frac{1}{1+10^{\left(p\mathrm{Ka}-{\mathrm{pH}}_{\text{Milk}}\right)}}$$

For a diprotic base:

(9) $$\begin{array}{c} f_{p}^{\;\mathrm{un}}=\frac{1}{1+10^{\left(\;p\mathrm{Ka}1+p\mathrm{Ka}2-\left(2*{\mathrm{pH}}_{\text{Plasma}}\right)\right)}+10^{\left(p\mathrm{Ka}1–{\mathrm{pH}}_{\text{Plasma}}\right)}+10^{\left(p\mathrm{Ka}2–{pH}_{\text{Plasma}}\right)}} \end{array}$$

(10) $$\begin{array}{c} f_{\mathrm{mk}}^{\;\mathrm{un}}=\frac{1}{1+10^{\left(\;p\mathrm{Ka}1+p\mathrm{Ka}2-\left(2*{\mathrm{pH}}_{\text{Milk}}\right)\right)}+10^{\left(p\mathrm{Ka}1-{\mathrm{pH}}_{\text{Milk}}\right)}+10^{\left(p\mathrm{Ka}2-{\mathrm{pH}}_{\text{Milk}}\right)}} \end{array}$$

For a monoprotic acid:

(11) $$f_{p}^{\;\mathrm{un}}=\frac{1}{1+10^{\left({\mathrm{pH}}_{\text{Plasma}}-p\mathrm{Ka}\right)}}$$

(12) $$f_{\mathrm{mk}}^{\;\mathrm{un}}=\frac{1}{1+10^{\left({\mathrm{pH}}_{\text{Milk}}-p\mathrm{Ka}\right)}}$$

For neutral compounds:

$$f_{p}^{\;\mathrm{un}}=1\;\text{and}\;f_{\mathrm{mk}}^{\;\mathrm{un}}=1$$

The calculated M/P ratio for each simulated subject was then used to predict milk exposure after maternal drug intake according to the following equation:

(13) $$\begin{array}{c} \text{Milk concentration}\;\left(\frac{\text{mass}}{\mathrm{vol}}\right)\\ =\frac{M}{P}\times \text{Maternal plasma concentration}\;\left(\frac{\text{mass}}{\mathrm{vol}}\right) \end{array}$$

#### 2.1.2 Calculation of Infant Dose

For all compounds and both models, the absolute IDD was calculated using the predicted average drug concentration in the milk for each simulated lactating woman ($\text{milk}\;C_{\mathrm{avg}}$), for each day assuming a constant milk intake of 0.15 L/kg/day by the infant [20, 21]. Many antimalarials are given as part of 3‐day treatment regimens. For comparison to the licensed pediatric dose, IDD was calculated based on $\text{milk}\;C_{\mathrm{avg}}$ on Day 3 to account for any accumulation. For the calculation of RID, IDD was calculated using $\text{milk}\;C_{\mathrm{avg}}$ for the whole duration of the trial to account for prolonged exposure.

(14) $$\text{Absolute}\;\mathrm{IDD}\;\left(\frac{\frac{\mathrm{\mu g}}{\mathrm{kg}}}{\mathrm{day}}\right)=\text{milk}\;C_{\mathrm{avg}}\;\left(\frac{\mathrm{\mu g}}{L}\right)\times \text{Daily Milk Intake}\left(\frac{\frac{L}{\mathrm{kg}}}{\mathrm{day}}\right)$$

RID was calculated using the absolute IDD, study duration and total maternal dose based on each clinical study.

(15) $$\mathrm{RID}\left(\%\right)=\frac{\text{Absolute}\;\mathrm{IDD}\;\left(\frac{\frac{\mathrm{\mu g}}{\mathrm{kg}}}{\mathrm{day}}\right)\times \text{study duration}\;\left(\text{days}\right)}{\text{maternal dose}\;\left(\frac{\mathrm{\mu g}}{\mathrm{kg}}\right)}\times 100$$

The maternal dose was the sum of all doses taken by the mother over the duration of the study per kilogram of maternal body weight.

#### 2.1.3 Lactation Simulation Design

All PBPK simulations were run in the Simcyp Simulator V21 (Certara UK Ltd., Sheffield). The M/P ratio models were incorporated into the Simulator using Lua scripting. The scripts are provided in the Supporting Information. Previously developed and verified PBPK models for the antimalarial compounds [22] were used without modification with the exception of piperaquine where the model was adapted as described in the Supporting Information.

#### 2.1.4 Antimalarial Drugs for Which Clinical Lactation Data Are Available (Model Verification)

The clinical trial design, availability of milk data (including pH and creamatocrit) and the virtual trial design for each drug are summarized in Table S1. Drug concentration‐time profiles were digitized from publications using GetData software. The virtual population demographics and trial size were matched to those reported for the clinical studies and 20 virtual trials were simulated for each clinical study (Table S1). When the trial size was smaller than 10 subjects, the virtual trial was set at a minimum size of 10. Where breastmilk characteristics were reported in the original studies, they were matched in simulations. In the absence of information, default milk physiological parameters for mature milk (i.e., milk pH = 7.2, creamatocrit = 6.2%) were assumed. Where available, the observed clinical pharmacokinetic profiles were compared to the predicted profiles.

#### 2.1.5 Antimalarial Drugs With No Clinical Lactation Data Available (Prospective Predictions)

Lactation PBPK modeling was applied to seven structurally diverse antimalarials, for which there were no clinical lactation data (amodiaquine and its active metabolite desethylamodiaquine, atovaquone, dihydroartemisinin, lumefantrine, proguanil, pyronaridine and tafenoquine).

Prospective predictions were performed using clinically relevant dosing regimens (summarized in Table S2) and a virtual trial design consisting of 200 virtual lactating women aged 18–45 years (20 trials of 10 subjects). Default parameters for mature milk (i.e., milk pH = 7.2, creamatocrit = 6.2%) were used for all prospective simulations.

### 2.2 Assessment of Model Performance

For the five antimalarials with clinical information, simulated mean, 5th, and 95th percentiles of plasma and milk concentration‐time profiles were compared with the observed data and their variability. Additionally, models were assessed using the average fold error (AFE) as a measure of bias and absolute average fold error (AAFE) and root mean squared error (RMSE) as a measure of precision as described below.

$$\mathrm{AFE}=10^{\frac{1}{n}\cdot \sum_{i=1}^{n}\log\left(\frac{\text{Predicted}}{\text{Observed}}\right)}$$

$$\text{AAFE}=10^{\frac{1}{n}\cdot \sum_{i=1}^{n}\left|\log\left(\frac{\text{Predicted}}{\text{Observed}}\right)\right|}$$

$$\text{RMSE}=\sqrt{\frac{1}{n}\cdot \sum_{i=1}^{n}{\left(\text{Predicted}-\text{Observed}\right)}^{2}}$$

where *n* = number of observations and *i* = individual value.

### 2.3 Sensitivity Analyses

The sensitivity of predicted M/P ratio to drug parameters and breastmilk characteristics was assessed using Model 1 and 2. For drug parameters, the relationships between predicted M/P ratio and Log*D*_7.2_ and fu were visualized using heatmaps based on an example basic drug with a *p*Ka of 9. The sensitivity of predicted M/P ratio to the breastmilk pH and creamatocrit was assessed across ranges of in vivo parameters reported in the literature [23, 24].

## 3 Results

### 3.1 Physicochemical Properties of the Investigated Drugs

The physicochemical properties of the investigated drugs are described in Table 1. The majority of these drugs were bases (10 out of 12) and all were lipophilic (Log*P* from 2.3 to 9.1, as reported previously [22]). They were generally highly bound to plasma proteins (fu from 0.001 to 0.4). The unbound fraction of the drug in skimmed milk calculated from fu ranged from 0.54 to 0.95.

**TABLE 1:** Physicochemical properties, protein binding and milk partitioning of the studied antimalarials.

Compound	Compound type	*p*Ka1	*p*Ka2	Log*P*_*o*:*w*_	Log*D*_7.2_	*P*_milk_	fu	fu_mk_
Chloroquine	Diprotic base	9.94	8.40	4.37	0.62	0.83	0.400	0.95
Mefloquine	Monoprotic base	8.53	NA	3.86	2.51	231	0.015	0.80
Primaquine	Diprotic base	10.20	3.30	3.13	0.54	0.66	0.260	0.93
Pyrimethamine	Monoprotic base	6.86	NA	2.52	2.36	145	0.095	0.90
Piperaquine	Diprotic base	8.80	7.40	5.27	3.26	2145	0.006	0.72
Atovaquone	Monoprotic acid	4.28	NA	8.40	5.51	1,711,268	0.001	0.54
Lumefantrine	Monoprotic base	9.80	NA	9.10	6.64	49,136,588	0.003	0.66
Tafenoquine	Diprotic base	8.74	6.00	5.57	4.01	19,391	0.005	0.71
Pyronaridine	Diprotic base	10.20	9.20	4.52	0.01	0.13	0.058	0.88
Amodiaquine	Diprotic base	7.59	7.04	3.51	2.74	458	0.089	0.90
Dihydroartemisinin	Neutral	NA	NA	2.30	2.30	122	0.105	0.90
Proguanil	Diprotic base	11.10	2.10	3.19	0.24	0.27	0.250	0.93

*Note: p*Ka, Log*P*_*o*:*w*_, Log*D*_7.4_, and fu values for all the listed drugs were published in Abla et al. [22].

Abbreviations: fu, fraction unbound in maternal plasma; fu_mk_, fraction unbound in skimmed milk (Equation 5); Log*D*_7.2_, logarithmic lipophilicity (calculated from Log*D*_7.4_); Log*P*_*o*:*w*_, logarithmic octanol–water partition coefficient; NA, non‐applicable; *p*Ka1, *p*Ka2, acid dissociation constants; *P*_milk_, milk‐lipid‐to‐ultrafiltrate partition coefficient (Equation 6).

### 3.2 Antimalarial Drugs for Which Clinical Lactation Data Are Available (Model Verification)

Clinical lactation studies were available for five antimalarial drugs: chloroquine, mefloquine, piperaquine, primaquine and pyrimethamine (Table S1).

**FIGURE 1:**
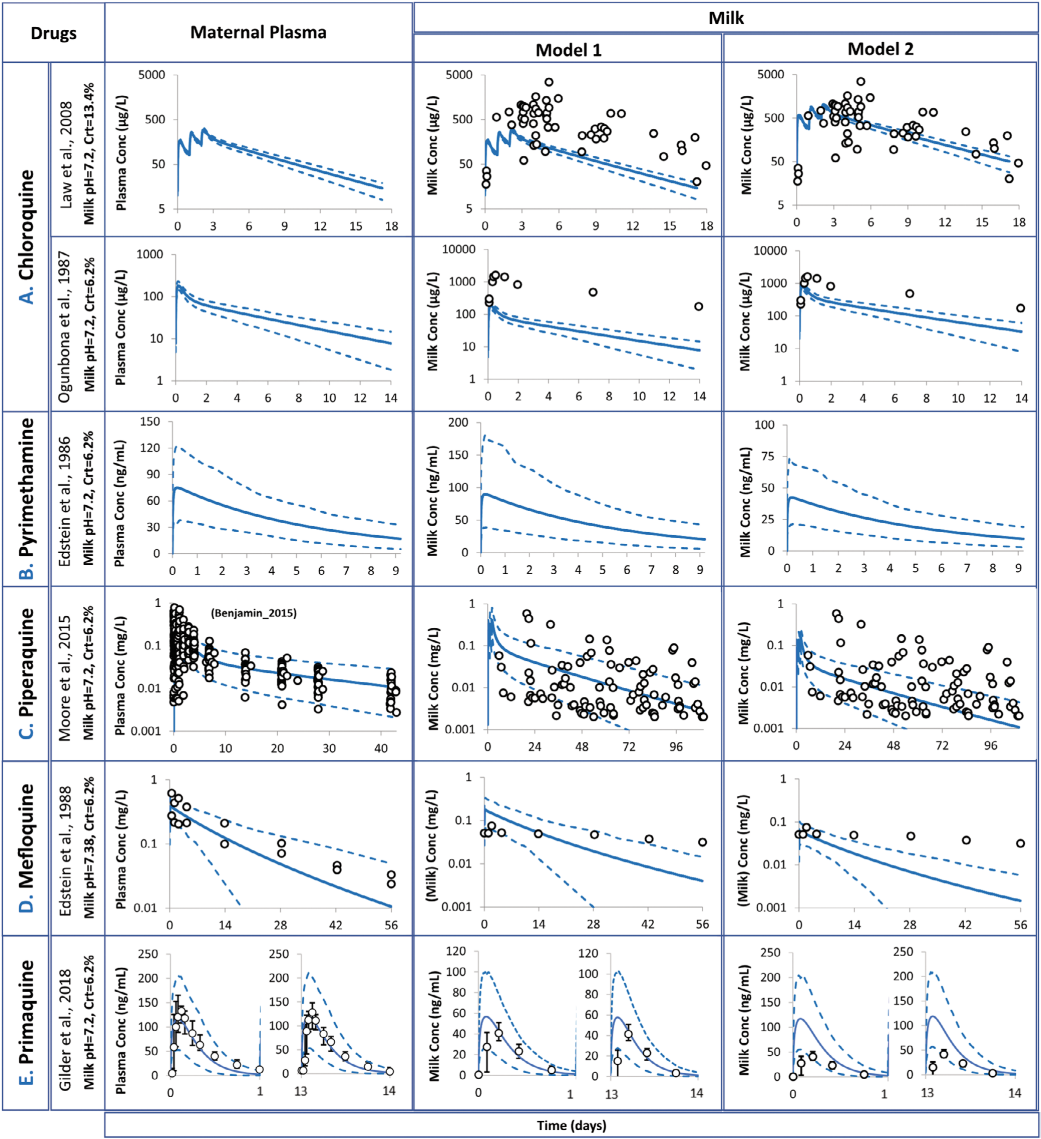
Simulated concentrations of five antimalarial drugs (A) Chloroquine; (B) Pyrimethamine; (C) Piperaquine; (D) Mefloquine; (E) Primaquine (verification set) in plasma (left) and in milk (right). Milk predictions are shown for both lactation models and overlaid with clinical data, where available (for trial design, refer to Table S1). For piperaquine, no clinical data were available in lactating women but observed PK data in non‐pregnant women were available from an independent study Benjamin et al. [25] and included here for comparison. Model 1, Phase distribution model; Model 2, Log‐transformed distribution model. Solid lines, Predicted means; Dashed lines, 5th and 95th percentiles; Circles, Observed data (Law, 2008 (*n* = 16) [26], Ogunbona, 1987 (*n* = 1) [27], Edstein, 1986 (*n* = 3) [28], Moore, 2015 [29], Edstein, 1988 (*n* = 2 subjects for plasma; *n* = 1 subject for milk) [30], Gilder, 2018 [31]).

The predicted PK profiles in plasma and milk were compared to those observed clinically (Figure 1) and the available observed and predicted M/P ratio, IDD and RID are described in Table 2.

**TABLE 2:** Predicted and observed pharmacokinetic parameters of antimalarial drugs in human milk.

Drug	Clinical study	Parameter	Observed	Predicted	
				Model 1	Model 2
Chloroquine	Law, 2008 (*n* = 16) [26] Milk pH: 7.2 (assumed) Creamatocrit: 13.4% (reported)	Milk/Plasma	NA	*0.99*	*3.46*
		Infant Daily Dose (μg/kg/day)	*34*	*12.3*	*43.0*
		Relative Infant Daily Dose (%)	*2.3*	*0.83*	*2.9*
	Edstein, 1986 (*n* = 3) [28] Milk pH: 7.2 (reported) Creamatocrit: 6.2% (assumed)	Milk/Plasma	2.86	1.01 ± 0.06	3.49 ± 0.18
		Infant Daily Dose (μg/kg/day)	9.39^2^	3.66 ± 0.71	12.6 ± 2.4
		Relative Infant Daily Dose (%)	1.83^2^	0.75 ± 0.16	2.6 ± 0.54
	Akintonwa, 1988 (*n* = 6) [32] Milk pH: 7.2 (assumed) Creamatocrit: 6.2% (assumed)	Milk/Blood Milk/Plasma	0.358 ± 0.007 1.25 ± 0.22^1^	0.33 ± 0.02 1.01 ± 0.06	1.13 ± 0.10 3.49 ± 0.18
		Infant Daily Dose (μg/kg/day)	NA	2.43 ± 0.60	8.37 ± 2.04
		Relative Infant Daily Dose (%)	NA	0.77 ± 0.19	2.67 ± 0.65
	Ogunbona, 1987 (*n* = 5) [27] Milk pH: 7.2 (reported) Creamatocrit: 6.2% (assumed)	Milk/Plasma	6.6 ± 2.4	1.01 ± 0.06	3.49 ± 0.18
		Infant Daily Dose (μg/kg/day)	NA	5.44 ± 1.18	18.8 ± 4.1
		Relative Infant Daily Dose (%)	NA	0.84 ± 0.20	2.9 ± 0.7
Pyrimethamine	Edstein, 1986 (*n* = 3) [28] Milk pH: 7.2 (reported) Creamatocrit: 6.2% (assumed)	Milk/Plasma	0.54	1.23 ± 0.46	0.58 ± 0.14
		Infant Daily Dose (μg/kg/day)	3.72^2^	7.09 ± 3.3	3.34 ± 1.3
		Relative Infant Daily Dose (%)	17.4^2^	33.3 ± 15.6	15.7 ± 6.1
Piperaquine	Moore, 2015 (*n* = 27) [29] Milk pH: 7.2 (assumed) Creamatocrit: 6.2% (assumed)	Milk/Plasma	*0.583*	*1.8*	*0.73*
		Infant Daily Dose (μg/kg/day)	*0.41*	*4.09*	*1.58*
		Relative Infant Daily Dose (%)	*0.004*	*1.30*	*0.50*
Mefloquine	Edstein, 1988 (*n* = 2) [30] Milk pH: 7.38 (reported) Creamatocrit: 6.2% (assumed)	Milk/Plasma	0.13^3^, 0.16^3^, 0.27^4^	0.46 ± 0.20	0.16 ± 0.05
		Infant Daily Dose_4days_ (μg/kg/day)	NA	21.8 ± 10.5	7.55 ± 2.54
		Relative Infant Daily Dose_4days_ (%)	3.45	2.4 ± 1.2	4.94 ± 1.95
		Infant Daily Dose_56days_ (μg/kg/day)	NA	5.7 ± 3.2	2.0 ± 0.93
		Relative Infant Daily Dose_56days_ (%)	2.6	8.7 ± 5.3	3.0 ± 1.6
Primaquine	Gilder, 2018 (*n* = 20) [31], Day 1 Milk pH: 7.2 (assumed) Creamatocrit: 6.2% (assumed)	Milk/Plasma	*0.34*	*0.48*	*0.99*
		Infant Daily Dose (μg/kg/day)	*2.98*	*2.76*	*5.64*
		Relative Infant Daily Dose (%)	*0.618*	*0.55*	*1.13*
	Gilder, 2018 (*n* = 20) [31], Day 14 Milk pH: 7.2 (assumed) Creamatocrit: 6.2% (assumed)	Milk/Plasma	*0.37*	*0.48*	*0.99*
		Infant Daily Dose (μg/kg/day)	*2.58*	*2.77*	*5.66*
		Relative Infant Daily Dose (%)	*0.517*	*0.55*	*1.13*

*Note:* Data presented as *median*, or mean ± standard deviation. Model 1, phase distribution model; Model 2, Log‐transformed distribution model. The duration of the study used in the calculation of relative infant daily dose—the percentage of maternal dose—was matched to that used in the clinical study for the same purpose. ^1^Converted from reported milk/blood ratio using a blood/plasma ratio of 3.5 for chloroquine, ^2^Calculated from observed milk AUC or C_avg_ provided in the publication, ^3^Individual value based on AUC_4days_, ^4^Individual value based on AUC_56days_.

Abbreviations: μg, microgram; kg, kilogram; *n*, number of lactating women in the study; NA, Not available.

**FIGURE 2:**
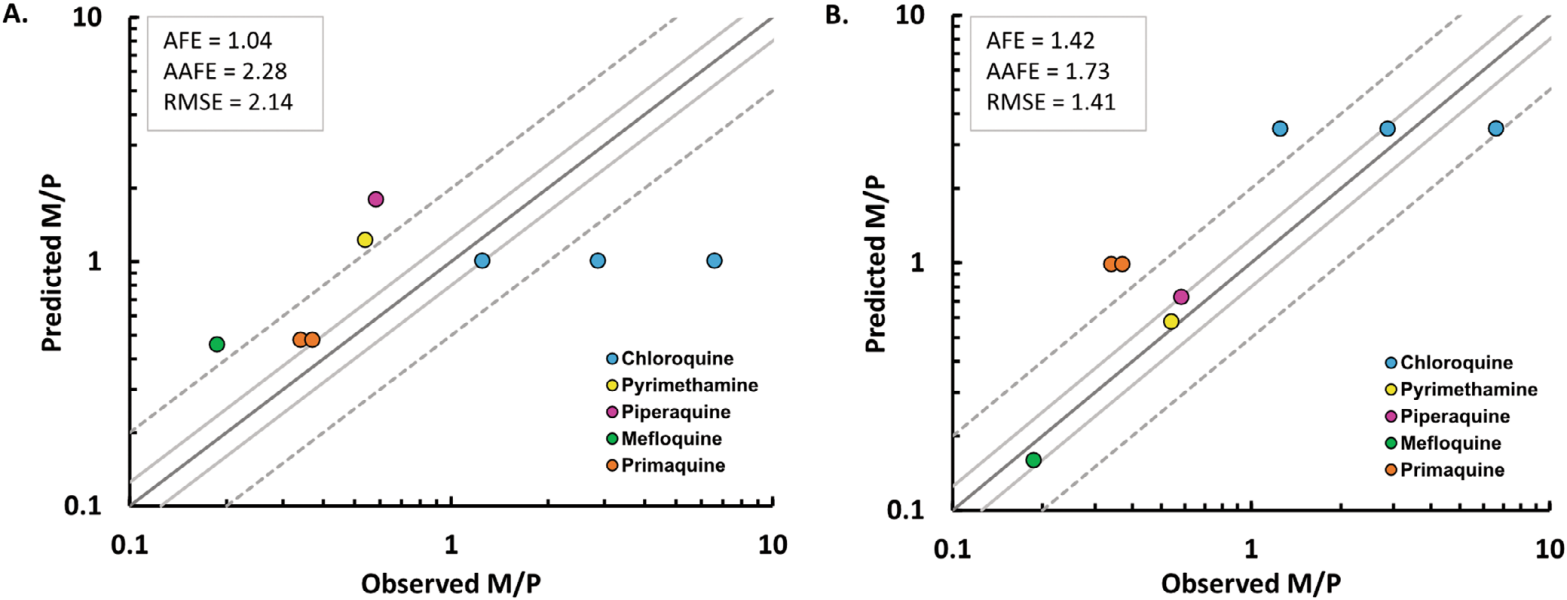
Predicted vs. observed M/P ratio for five antimalarials (verification set) using (A) Model 1: Phase distribution model, and (B) Model 2: Log‐transformed distribution model. Gray solid lines and dashed lines represent 1.25‐fold and 2‐fold predictions, respectively. AAFE, Absolute average fold error; AFE, Average fold error; RMSE, Root mean squared error.

Overall M/P ratio prediction accuracy had a bias (AFE) and a precision (AAFE and RMSE) of 1.04, 2.28 and 2.14, using the phase distribution model (Model 1), and 1.42, 1.73 and 1.41, using the log‐transformed model (Model 2) (Figure 2). For Model 1, 75% of predicted M/P ratios were within 3‐fold of observed, with the majority (63%) within 2‐fold. For Model 2, all predicted M/P ratios were within 3‐fold of observed, with the majority (63%) within 2‐fold.

The RID was below 10% for all drugs except for pyrimethamine. Both methods correctly predicted whether the RID was above or below the WHO cut‐off of 10% (Table 2).

### 3.3 Prospective Lactation PBPK Model Predictions for Antimalarials With No Clinical Information

Prospective PBPK predictions of M/P ratios, IDD and corresponding RID for seven antimalarials for which there are no clinical lactation data are presented in Table 3.

**TABLE 3:** Prospective predictions of exposure to antimalarials from breastmilk assuming 150 mL/kg/day milk intake (with no clinical lactation data).

Compound	Milk/plasma		Absolute infant dose (mg/kg/day)		Relative infant dose (%)		Infant dose for malaria treatment/prophylaxis (age/weight range)
	Model 1	Model 2	Model 1	Model 2	Model 1	Model 2	
Amodiaquine	4.17	1.92	0.005	0.003	0.07	0.03	75 mg QD for 3 days (3–< 12 months) [33]
Desethylamodiaquine	0.64	1.52	0.253	0.06	NC	NC	
Atovaquone	66.2	<< 0.00001	80.7	<< 0.00001	1203	<< 0.00001	125 mg QD for 3 days (5–8 kg) [34]
Dihydroartemisinin	1.05	NA	0.005	NA	0.20	NA	20 mg artesunate (15 mg DHA) QD for 3 days (5–< 8 kg) [35]
Lumefantrine	> 10,000	33.4	22,535	51.3	> 200,000	485	120 mg BID for 3 days (5–< 15 kg) [36] Doses for babies 2–< 5 kg under investigation in a clinical study [37]
Proguanil	0.45	0.95	0.012	0.026	0.24	0.50	50 mg QD for 3 days (5–8 kg) [34]
Pyronaridine	0.18	0.78	0.005	0.023	0.23	1.00	60 mg (34 mg free base) QD for 3 days (5–< 8 kg) [35]
Tafenoquine	9.59	0.98	0.277	0.026	111	11.2	Not indicated for infants < 2 years

*Note:* Values in the table are presented as mean. Model 1, phase distribution model; Model 2, Log‐transformed distribution model.

Abbreviations: BID, *bis in die*, twice a day; DHA, Dihydroartemisinin; NA, Not available, Method 2 is not available for neutral drugs; NC, not calculated for metabolites; QD, *quaque die*, once a day.

Using a default milk pH of 7.2 and a creamatocrit of 6.2%, predicted M/P ratio for amodiaquine (desethylamodiaquine), proguanil, pyronaridine and tafenoquine were 4.17 (0.64), 0.45, 0.18 and 9.56 using Model 1 and 1.92 (1.52), 0.95, 0.78, and 0.98 using Model 2, respectively. The IDD for amodiaquine (desethylamodiaquine), proguanil, pyronaridine and tafenoquine were 0.005 (0.253), 0.012, 0.004 and 0.277 mg/kg/day using Model 1 and 0.003 (0.06), 0.026, 0.023 and 0.026 mg/kg/day using Model 2. Using these data and assuming a daily milk intake of 150 mL/kg, the predicted RID using the average milk concentrations for amodiaquine, proguanil, pyronaridine and tafenoquine were 0.07%, 0.24%, 0.23% and 111% using Model 1 and 0.03%, 0.50%, 1.00% and 11.2% using Model 2, respectively (Table 3). Although different in magnitude, the RID for tafenoquine was > 10% using both models.

For atovaquone, Model 1 predicted a very high M/P ratio of 66.2 with corresponding IDD and RID of 80.7 mg/kg/day and 1203%, respectively, whereas Model 2 predicted a low M/P ratio, IDD and RID (< 0.00001). For lumefantrine, Model 1 predicted extremely high values (> 10,000 for M/P ratio, IDD and RID), and Model 2 predicted a high M/P ratio of 33.4 with an IDD and RID of 51.3 mg/kg/day and 485%, respectively. Lastly, dihydroartemisinin predictions were only performed with Model 1 (M/P ratio of 1.05 with RID of 0.2%) as Model 2 is not applicable for neutral compounds (Table 3).

### 3.4 Assessment of the Sensitivity of M/P Ratio to Changes in the Drug Properties fu and Log*D*_7_ _.2_

**FIGURE 3:**
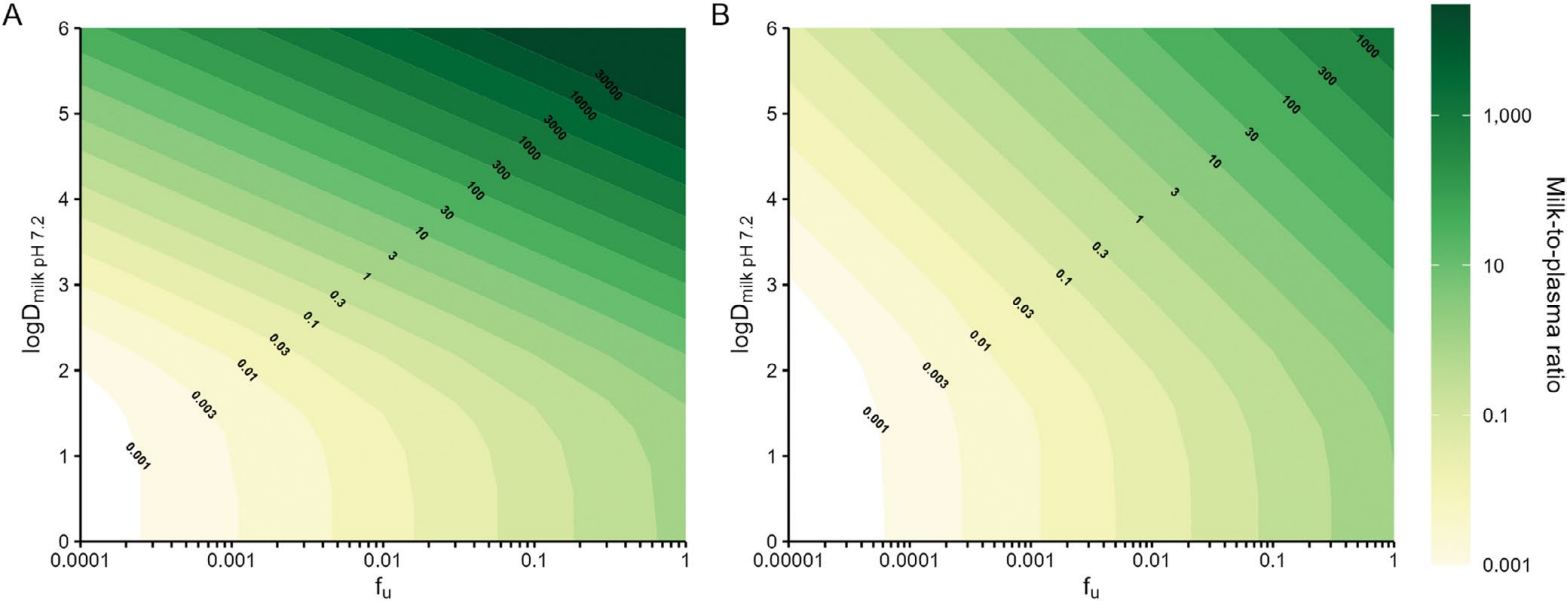
Heatmaps showing the relationship among fu and Log*D*_7.2_ and M/P ratio (contours) calculated using (A) Model 1 and (B) Model 2 for a basic drug (*p*Ka = 9).

The relationship between M/P ratio and the drug properties Log*D*_7.2_ and fu for a basic drug (with *p*Ka of 9) is shown in Figure 3A (Model 1) and 3B (Model 2). For both models there was a trend towards higher M/P ratio with higher fu and Log*D*_7.2_. For this example drug, when fu was equal to 1, M/P ratio was greater than 1 even for the lowest Log*D*_7.2_. When Log*D*_7.2_ was equal to or > 4.5, M/P ratio was 1 or greater, even when fu was 0.0001. Both Log*D*_7.2_ and fu were sensitive parameters for the calculation of M/P ratio.

### 3.5 Assessment of the Sensitivity of M/P Ratio to Variability in Breastmilk Creamatocrit and pH

**FIGURE 4:**
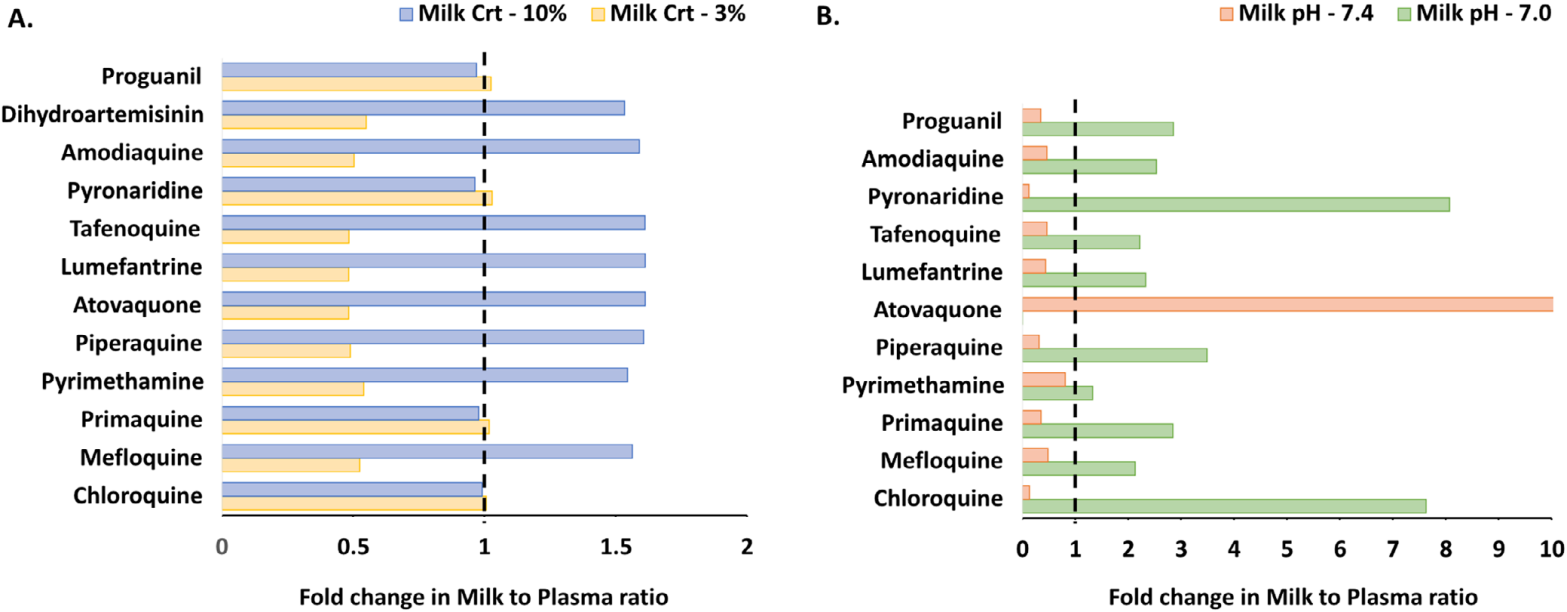
(A) Sensitivity of milk to plasma ratio prediction to milk creamatocrit (Crt) using Model 1 (method with the greatest sensitivity to fat composition), (B) Sensitivity of milk to plasma ratio prediction to milk pH in Model 2 (method with the greatest sensitivity to milk pH). Black dashed line represents no fold change. Fold change in milk to plasma ratio is relative to default milk creamatocrit composition (6.2%) and milk pH (7.2).

Predicted M/P ratio was more sensitive to creamatocrit than pH for Model 1 and *vice versa* for Model 2. The fold change in M/P ratio when the creamatocrit and pH are changed from default (most typical) values to the highest and lowest reported values are presented in Figure 4. Using Model 1, the M/P ratio for 8/12 drugs varied ± 2‐fold when creamatocrit was either increased from 6.2% to 10% or reduced to 3%. Using Model 2, the M/P ratio for all drugs apart from pyrimethamine (base) changed more than 2‐fold when breastmilk pH values were varied across the range of those observed [23, 24]. Dihydroartemesinin was not included in this part of the sensitivity analysis as method 2 is not usable for neutral compounds.

## 4 Discussion

Although breastfeeding is recommended [2, 3] and the norm in malarial endemic regions [4], label recommendations to avoid breastfeeding while using antimalarial drugs are still inconsistent across different regulatory regions (Table S3). The WHO guidelines for malaria recommend using artemisinin‐based combination therapies in lactating women but this recommendation is not based on strong evidence. While recent regulatory guidance requires drug developers to consider population diversity in clinical trial design [10] and labeling rules require drug labels to summarize the risks of drug presence in the breastmilk and its potential effect on the breastfed infant for new drugs [38], lactating mothers remain an understudied population and the assessment of all current antimalarials in clinical lactation studies is challenging due to operational, time and cost constraints. Here we used a PBPK approach to predict dynamic concentrations in milk, using M/P ratio calculated from physicochemical and maternal (breastmilk) characteristics, and propose it as a practical approach to evaluate whether clinical evaluation is warranted. This approach may also be helpful to inform the necessity of breastfeeding‐related study exclusion criteria for Phase III studies.

This work first assessed the prediction of milk exposures of five antimalarials for which clinical data were available (verification dataset) and prospectively predicted the concentrations for an additional seven drugs for which no clinical lactation data are available.

The current WHO RID classification approach uses an arbitrary cut‐off value of RID < 10% [20] to indicate theoretical compatibility with dosing to breastfeeding mothers [39, 40]. Regardless of the model selected (Model 1 vs. Model 2), the predicted RID for all drugs in the verification dataset was correctly categorized as above or below 10%. With the exception of pyrimethamine, the RID were predicted to be < 10%. This suggests minimal distribution of these drugs to human milk, and hence limited safety risks in infants based on low milk exposure. Therefore, despite the differences between the two models, the overall classification of the compounds according to the WHO criterion was considered acceptable. Predictions of M/P ratio were within 3‐fold of clinical observations in all studies (100%) for Model 2 and in 75% of studies for Model 1 (Figure 2). Overall, the log transformed model (Model 2) performed better (based on calculated AFE, AAFE and RMSE) compared to the phase distribution model (Model 1).

For the prospective simulations, the two methods gave the same assessment of RID being below or above 10% for 5/6 drugs tested with both models. As dihydroartemisinin is a neutral compound, only Model 1 was used and predicted a very low RID of ~0.2%. Amodiaquine, proguanil and pyronaridine also had predicted RID below 1%, suggesting that breastfed infants should have low exposures to these drugs. Lumefantrine had a RID above 10% using both methods. The high predicted RID suggests lumefantrine partitioning into milk could be considerable but at the same time this extreme value may question the applicability of the models for compounds as lipophilic as lumefantrine (see below). A clinical lactation study with artemether and lumefantrine is currently ongoing and will shed light on the actual M/P ratio for lumefantrine [41]. Tafenoquine also had a predicted RID > 10% with both models. For atovaquone, the predicted RID was > 10% with Model 1 but < 10% for Model 2. This makes the interpretation of the results for this compound more difficult, especially since the RID predicted using Model 1 is particularly high.

Model performance should be interpreted in the context of the model limitations for compounds with physicochemical properties outside that of the original QSAR model training dataset. The available QSAR models were developed with 14 compounds (6 acids and 8 bases) with lipophilicity ranging from −0.29 to 3.11 and protein binding ranging from negligible to 99.9% (only 4 drugs with > 98.8% binding) [42], to determine milk lipid‐to‐ultrafiltrate partition coefficient ($P_{\text{milk}}$) and fraction unbound in milk, respectively. Selection of the appropriate method (Model 1 vs. 2) for prospective M/P ratio predictions is challenging as the two models are likely to impact drugs with different properties differently. For example, Model 1 attributes equal weighting to the variables considered in the model (lipophilicity, ionization, protein binding), whereas Model 2 gives less weight to lipophilicity and protein binding compared to ionization (Equations (1), (2), (3)). Thus, it may be expected that at low milk pH (i.e., < 7.0), Model 2 may overestimate M/P ratio for basic drugs with high *p*Ka while Model 1 may overestimate M/P ratio for basic drugs with very low plasma fu (< 0.01) and high lipophilicity. Clearly, a much larger number of drugs are required to ascertain the optimal method for accurate M/P ratio prediction for a drug a priori, but this exercise is already an important step towards informing clinical studies.

The properties of drugs for which prospective predictions were carried out were compared to those used in the verification data set and those originally used to develop the QSAR models. As is typical in the antimalarial drug space, the drugs used for prospective predictions in this analysis were lipophilic (LogP ranging from 2.3 to 9.10) and 10/12 were basic (1 acid and 1 neutral) and were generally highly bound to plasma proteins (fu ranged from 0.001 to 0.4 (Table 1)) with four of the drugs having an fu < 0.01. Particularly, atovaquone, tafenoquine and lumefantrine are highly lipophilic (Log*P* = 8.40, 5.6 and 9.1 respectively) and highly protein bound (fu = 0.001, 0.005 and 0.003 respectively). Not only do they deviate markedly from the range of Log*P* values (−0.29 to 3.11) for drugs used to derive the prediction equation for *P*_milk_ [43], these Log*P* values are also greater than the lipophilic antimalarials used for model verification in our study (2.5 to 5.3). Furthermore, the acidic properties of atovaquone may be the cause of the low value using Model 2.

Understanding the impact of drug physicochemical (*p*Ka and log*D*) and binding (fu) properties on the M/P ratio predictions can prioritize additional milk‐related in vitro measurements of key parameters for compounds where the predictions can be highly sensitive to such parameters.

The relationship between M/P ratio and the drug parameters fu and Log*D*_7.2_ for a basic drug defined using Model 1 and 2 were illustrated using heatmaps. A basic *p*Ka of 9 was selected as the majority of antimalarials tested were basic and had an average *p*Ka of around 9. As expected, the examples show the trend for higher M/P ratio with higher Log*D*_7.2_ and fu. The heatmaps provide a visual reference for the likely M/P ratio for a basic drug once these parameters are known. They also show the sensitivity of M/P ratio to fu and Log*D*_7.2_ highlighting the importance of robust estimates. The use of sensitive (e.g., for low fu) and experimental determinations (e.g., measured versus calculated Log*D*) may be helpful here. It should also be noted that error in determination of fu is propagated into the prediction of fu_mk_, especially at fu < 0.05 where small changes can lead to large differences in fu_mk_ [42].

Additionally, the partitioning of drug between whole and skimmed milk (S/W) is a key component in Model 1 that is highly sensitive to Log*D*_7.2_ and can be determined experimentally [17]. The optimal approach would be to measure fu_mk_ when fu is < 0.05 (i.e., > 95% protein binding) and to measure the skimmed to whole milk partitioning (S/W) or *P*_milk_ when Log*D*_7.2_ is > 3. Currently, such in vitro assays are not routine and finding a source of human milk with characterized composition (e.g., depending on the age of the infant) is challenging.

Understanding the impact of maternal variables, such as change in the milk pH and creamatocrit, on drug concentrations in milk can aid optimal interpretation of clinical M/P ratio data in the context of interindividual variability, particularly where parameters such as pH or creamatocrit have not been measured. Here, we focused on milk pH and creamatocrit and selected the model with the highest sensitivity for each milk parameter. Hence, Model 1 and Model 2 were used to identify the drugs for which M/P ratio is sensitive to creamatocrit and milk pH, respectively. M/P ratios for 8/12 drugs tested were sensitive to creamatocrit when a percentage of fat of 3% and 10% (~0.5 and 1.5‐fold of average creamatocrit, respectively) were compared. Except for pyrimethamine (*p*Ka of 6.86), M/P ratio was sensitive to pH for all basic drugs (*p*Ka of 7.6 to 11.1). Milk pH varies between individuals and is reported to decrease in the same individual during the first postpartum month [43, 44]. Therefore, for antimalarial drugs which are rather lipophilic, measuring milk pH and creamatocrit in clinical lactation studies could allow a better understanding of the clinical results despite the logistical burden of additional measurements. The sensitivity analyses also highlight the potential impact on model performance assessments when the available clinical lactation studies do not report these data. Equally, it is key to understand the input parameters defining milk composition when comparing model performance from different sources.

Of the assumptions made in this analysis the most significant was that the transfer of drug to the milk is rapid. This assumption is reasonable in the absence of any known rate‐limiting transporter processes involved in transfer of the studied compounds to milk. A recent review of the literature [45] identified the lactational mRNA and/or protein expression of 20 drug transporters and highlights the need to understand the relevant transporter mechanisms for the drug of interest. Another limitation of this analysis is that a constant infant daily milk intake and the static milk composition were used for all subjects, without accounting for interindividual variability or longitudinal changes in milk composition [13]. The results of sensitivity analysis assessing the impact of creamatocrit and milk pH can be used to inform the likely impact of changes in milk composition and interindividual variability for each drug.

For most antimalarials tested here, doses are available for treatment and/or prophylaxis of infants weighing more than 5 kg and have an adequate benefit/risk profile in infants (Table 3). This provides additional information to support decisions around the safety of breastfeeding. As recommended for data measured in clinical lactation studies [46], these licensed dosages for approved pediatric indications can provide context to the predicted IDD and its uncertainty, especially when the two models are not aligned.

The work presented here was done on marketed antimalarials, and moving forward, it would be important to collect information related to milk exposure as soon as possible for new molecules intended for treatment or prevention so to give guidance through labeling recommendations to the mother, the healthcare providers and the policy makers for newborns (including premature babies and infants). For such new drugs, PBPK will play a critical role to inform on the clinical data that would need to be collected in addition if at all needed. Close collaboration with regulatory authorities will be needed to firmly integrate the PBPK models into drug development for lactating women [47].

Once clinical lactation data are available to verify a model, the model can be extended to simulate the drug exposure in breastfed infants, taking developmental changes in infant enzyme expression (ontogeny) and physiological parameters into account. Variations in feeding patterns and spacing between drug administration and feeding may also be assessed. This approach was used to supplement recent clinical studies with primaquine [16, 31], adding to the weight of evidence supporting the use of primaquine in all breastfeeding women to treat the relapse of vivax malaria infections [48]. Tafenoquine, a more recent single dose radical cure for vivax malaria, has a predicted RID > 10%, with high variability between the two models. Furthermore, it is not currently indicated for infants < 2 years. In this case, a clinical lactation trial with tafenoquine would be key to provide confidence in the modeling and support the decision whether to administer tafenoquine to breastfeeding mothers. Based on our analyses and our aim to select compounds with low milk exposure, pyronaridine‐artesunate and dihydroartemisinin‐piperaquine were identified as potential candidates that warrant consideration for clinical lactation studies as the predicted IDD for pyronaridine, dihydroartemisinin and piperaquine suggests they are suitable for lactating women. Ideally, generating clinical lactation data for these two highly prescribed artemisinin‐based combination therapies would verify the breastmilk predictions which could then be used as inputs into simulations that predict the drug concentration in the infant. Simulations can then be used to supplement clinical lactation data, for example simulating a range of infant ages in addition to those recruited into a study.

In conclusion, we propose a practical strategy to evaluate whether a clinical lactation study is warranted and to inform drug labels or policy recommendations for new or older antimalarials. Firstly, PBPK modeling using two published QSAR models (here defined as Model 1 and 2) can be used to predict dynamic concentrations in milk and plasma over time. IDD can then be calculated using the average milk concentration on the third day of 3‐day regimens to account for potential accumulation. Where possible, the predicted IDD should be compared with dosages licensed for administration to < 1‐year‐olds to build confidence in the modeling and infer safety. With predicted IDD, RID can also be calculated to give an easy comparison to the WHO recommended 10% cut‐off. Discrepancies between Model 1 and 2, especially those impacting interpretation should be interrogated considering the robustness of experimentally determined parameters and whether additional in vitro data need to be generated. Finally, we recommend the impact of variability in breastmilk characteristics (e.g., pH and creamatocrit) should be considered to predict interindividual variability in drug exposure in milk and to assess a need for additional measurement in clinical lactation studies to aid interpretation.

Although the focus here is the treatment and prevention of malaria in lactating women, the strategy may also be considered for other indications where neither delaying treatment nor abstaining from breastfeeding are viable options.

## Author Contributions

L.M.A., A.P., B.K., I.G., A.C.M. and N.A. wrote the manuscript. L.M.A., K.A., A.P., H.M.J., K.R.Y., M.E.G., J.J.M., and N.A. designed the research. L.M.A., K.A., A.P., and N.A. performed the research. L.M.A., K.A., A.P., B.K., M.F. and N.A. analyzed the data.

## Ethics Statement

The authors have nothing to report.

## Conflicts of Interest

L.M.A., K.A., A.P., H.M.J., K.R.Y., I.G. and M.F. are employees of Certara Predictive Technologies Division and may hold shares in Certara Inc. B.K., A.C.M., M.E.G. and N.A. are employees of MMV Medicines for Malaria Venture. At the time of research, M.F. was an employee of Certara and J.J.M. was an employee of MMV Medicines for Malaria Venture. As Deputy Editor in Chief of *CPT: Pharmacometrics & Systems Pharmacology*, Karen Rowland Yeo was not involved in the review or decision process for this paper.

## Supporting information


**Data S1.** Supporting Information
**Data S2.** Lua script: Lactation log transformed distribution model base.
**Data S3.** Lua script: Lactation phase distribution model acid base neutral.
**Data S4.** Lua script: Lactation log transformed distribution model acid.

## Data Availability

The PBPK models used in this paper can be downloaded from the Global Health Repository following a registration step PBPK Repository – PBPK Repository (certara.co.uk).
