# Supplementary material for: PBPK‐Led Assessment of Antimalarial Drug Concentrations in Breastmilk: A Strategy for Optimal Use of Prediction Methods to Guide Decision Making in an Understudied Population
Source: CPT Pharmacometrics Syst Pharmacol. 2025 Feb 11;14(4):738–50. doi: 10.1002/psp4.13311 (PMC12001273; doi:10.1002/psp4.13311)
Supplement: Supplementary file 1 — Data S1. Supporting Information Data S2. Lua script: Lactation log transformed distribution model base. Data S3. Lua script: Lactation phase distribution model acid base neutral. Data S4. Lua script: Lactation log transformed distribution model acid. [file PSP4-14-738-s001.zip › psp413311-sup-0001-DataS1.docx]

**Supplemental Material**

**PBPK-led assessment of antimalarial drug concentrations in breastmilk: a strategy for optimal use of prediction methods to guide decision making in an understudied population**

Lisa M. Almond^1^, Khaled Abduljalil^1^, Amita Pansari^1^, Beata Kusmider^2^, Hannah M. Jones^1^, Karen Rowland-Yeo^1^, Iain Gardner^1^, Muhammad Faisal^1^, Anne Claire Marrast^2^, Myriam El Gaaloul^2^, Jörg J. Möhrle^2^ and Nada Abla^2#^

^1^ Certara Predictive Technologies, Simcyp Division, Sheffield, UK.

^2^ Medicines for Malaria Venture, Geneva, Switzerland.

^#^Corresponding author: Nada Abla, MMV Medicines for Malaria Venture

# Table S1. Clinical lactation data for selected antimalarial compounds

| **Drug** | **Study** | **General Trial Design** | | **Milk Data Availability** | **Virtual trial design** |
| --- | --- | --- | --- | --- | --- |
|  |  | **Population** | **Dosage/sampling** |  |  |
| **Chloroquine** | Law, 2008  (1) | 16 Melanesian (Papuan) lactating women  Age: 24 (22 - 27.5) years  Weight: 54 (51 - 57) kg  Postpartum time: 1 -3 days. | Dose: 465 mg base (in 750 mg phosphate salt) daily for three consecutive days  Sampling duration: 17.2 (16.1 – 18.1) days  Assay: HPLC | Milk pH: not reported  Creamatocrit: 13.4% (8.6 -18.9%)  Milk PK parameters  Milk (no plasma) concentration profiles | Age: 22 - 28 years  Trial: 20 x 16 subjects  Dose: 8.5 mg/kg/day (base) PO for 3 days  Milk pH: 7.2  Creamatocrit: 13.4% |
|  | Edstein, 1986  (2) | 3 presumed Australian lactating women  Age: 28 - 34 years  Weight: 60 - 67 kg  Postpartum time: within 2 - 5 days  Study duration: 9 - 9.5 days | Dose: single dose of 300 mg (base) PO  Blood & milk sampling:  Subject 1: 0, 4, 29, 53, 124 and 217h  Subject 2: 0, 4, 28, 53, 102 and 227h  Subject 3: 0, 4, 29, 52, 148 and 219h  Assay: HPLC | Milk pH: 7.19 (6.75 - 7.53)  Creamatocrit: not reported  Plasma and milk AUC  No PK profile was reported | Age: 28 - 34 years  Trial: 20 x 10 subjects  Dose: 300 mg (base) PO  Milk pH: 7.2  Creamatocrit: 6.2% |
|  | Akintonwa, 1988 (3) | 6 Nigerian lactating women  Age: 17 – 35 years  Postpartum time: 17 days | Dose: 3.1 mg/kg (base) as a single intramuscular injection  Sampling: 2h after administration  Assay: Reversed phase HPLC | Milk pH: not reported  Creamatocrit: not reported  M/P was determined at 2h after drug administration  No PK profile was reported | Age: 28 - 34 years  Trial: 20 x 10 subjects  Dose: 3 mg/kg (base) IV  Milk pH: 7.2  Creamatocrit: 6.2% |
|  | Ogunbona, 1987 (4) | 11 Nigerian lactating women  Postpartum time: not reported | Dose: single dose of 600 mg (base) PO  Sampling: at 0, 3, 6, 9, 12, 24, 48h and a few samples within 7 days  Assay: Reversed phase HPLC | Milk pH at 24h: 7.2±0.3, range: 6.9-7.9  Creamatocrit: not reported  Saliva and milk profiles from one subject  M/P (n=5) & M/S (n=6) for samples collected 24h after a single dose | Age: 18 - 45 years  Trial: 20 x 11 subjects  Dose: 600 mg (base) PO  Milk pH: 7.2  Creamatocrit: 6.2% |
|  | Ette, 1987 (5) | 5 Nigerian lactating women  Age: 28 (21 – 35) years  Weight: 62 (58 – 70) kg  Postpartum time: (60 – 90) days  All subjects had residual  levels of chloroquine at t=0.  Dosing history was not reported | Dose: single dose of 300 mg (base) PO  Duration: 7 days  Sampling: 0, 1, 3, 6, 9, 12, 24, 48, 72, 96, 120, 144, 168 h  Assay: thin layer chromatography/ spectrometry | Milk pH: 7.2±0.2,  Creamatocrit: not reported  Mean profile of chloroquine in milk, M/S  T_max_, AUC_INF_, t_1/2_, and CL/F in milk and saliva are not significantly different | Not simulated due to absence of information on dosing |

**Table S1.** Continued

| **Drug** | **Study** | **General Trial Design** | | **Milk Data Availability** | **Virtual trial design** |
| --- | --- | --- | --- | --- | --- |
|  |  | **Population** | **Dosage/sampling** |  |  |
| **Pyrimethamine** | Edstein, 1986  (2) | 3 presumed Australian lactating women  Age: 28 - 34 years  Weight: 60 - 67 kg  Postpartum time: within 2 - 5 days  Study duration: 9 - 9.5 days | Dose: Single dose of 12.5 mg PO  Blood & milk samples collection:  Subject 1: 0, 4, 29, 53, 124 and 217h  Subject 2: 0, 4, 28, 53, 102 and 227h  Subject 3: 0, 4, 29, 52, 148 and 219h  Assay: HPLC | Milk pH: 7.19 (6.67 - 7.53)  Creamatocrit: Not reported  Plasma and milk AUC  No PK profile was reported | Age: 28 - 34 years  Trial: 20 x 10 subjects  Dose: 12.5 mg (base) PO  Milk pH: 7.2  Creamatocrit: 6.2% |
| **Piperaquine** | Moore, 2015 (6) | 27 Melanesian (Papuan) lactating women  Age: 27±7 years  Weight: 52.4±7.1 kg  Subjects were enrolled and received treatment during pregnancy  Study duration: 125 days | Dose: 554 mg (base) PO for 3 days  Blood & milk sampling: At delivery, days 1, 2, 3 to 5, 7 to 11, and 14 to 17 postdelivery  Time from the first dose of breast milk collection: 70 days  Assay: HPLC | Milk pH: Not reported  Creamatocrit: Not reported  Milk (no plasma) concentration profiles only | Age: 18 - 45 years  Trial: 20 x 27 subjects  Dose: 11.53 mg/kg/day (base) PO for 3 days  Milk pH: 7.2  Creamatocrit: 6.2% |
| **Mefloquine** | Edstein, 1988  (7) | 2 Caucasian lactating women  Age: 26 and 30 years  Postpartum time: 2 - 3 days  Study duration: 56 days | Dose: a single oral dose of 250 mg mefloquine base | Milk pH: 7.38 (7.10 -7.80)  Creamatocrit: not reported  PK parameters  Plasma and milk concentration profile from one subject | Age: 26 - 30 years  Trial: 20 x 10 subjects  Dose: 250 mg (base) PO  Milk pH: 7.38  Creamatocrit: 6.2% |
| **Primaquine** | Gilder, 2018  (8) | 20 lactating women from Myanmar  Age: 18 - 40 years  Postpartum time: 1.5 - 22 months  Study duration: 14 days | Dose: multiple oral doses of 0.5 mg base/kg once daily for 14 days | Milk pH: Not reported  Creamatocrit: not reported  Plasma and milk PK parameters  M/P was calculated for primaquine  Plasma and milk concentration profiles | Age: 18 - 40 years  Trial: 20 x 20 subjects  Dose: 0.5 mg/kg/day (base) PO for 14 days  Milk pH: 7.2  Creamatocrit: 6.2% |

HPLC - high-performance liquid chromatography; PO - *per os,* oral; IV - intravenous; PK - pharmacokinetic; M/P - milk-to-plasma ratio; M/S - milk-to-saliva ratio; AUC - area under the curve, AUC_INF_ - total area under the curve from time 0 to infinity; T_max_ - time of maximum concentration; t_1/2_ - elimination half-life; CL/F - apparent oral clearance; mg - milligram; kg - kilogram; h - hour.

# Table S2. Dosing regimens used in prospective predictions

| **Drug** | **Dose (mg)** | **Interval (h)** | **Total number of doses** |
| --- | --- | --- | --- |
| Amodiaquine | 540 | 24 | 3 |
| Lumefantrine | 480 | Unequal intervals^#^ | 6 |
| DHA (dosed as artesunate) | 177.5* | 24 | 3 |
| Pyronaridine | 720 (410 free base) | 24 | 3 |
| Proguanil | 400 (350 free base) | 24 | 3 |
| Atovaquone | 1000 | 24 | 3 |
| Tafenoquine | 300 | N/A | 1 |
| ^#^First dose (0h) then 8, 24, 36, 48 and 60h after the first dose; *corresponding to a 240 mg dose of artesunate  DHA - dihydroartemisinin | | | |

# Table S3. Examples of the current status of labels and recommendations on the use of antimalarials during lactation

| **Drug** | **Organization** | **Labelling** |
| --- | --- | --- |
| Chloroquine | FDA | Application number: ANDA040516, updated 2022 (9)  Because of the potential for serious adverse reactions in nursing infants from chloroquine, a decision should be made whether to discontinue nursing or discontinue the drug, taking into account the potential clinical benefit of the drug to the mother. The excretion of chloroquine and the major metabolite, desethylchloroquine, in breast milk was investigated in eleven lactating mothers following a single oral dose of chloroquine (600 mg base). The maximum daily dose of the drug that the infant can receive from breastfeeding was about 0.7% of the maternal start dose of the drug in malaria chemotherapy. Separate chemoprophylaxis for the infant is required. |
|  | BNFC | Present in breast milk and breastfeeding should be avoided when used to treat rheumatic disease. Amount in milk is probably too small to be harmful when used for malaria (10). |
|  | WHO | Compatible with breastfeeding (11). Monitor for side effects (hemolysis and jaundice), especially if the infant is premature or less than 1 month old. Avoid in G6PD-deficient infants (12). |
|  | CDC | Chloroquine may be prescribed safely to infants; it is also safe for infants to be exposed to the small amounts excreted in breast milk. Pediatric dose: 5 mg/kg base (8.3 mg/kg salt) orally (13). |
| Pyrimethamine  (+ sulfadoxine) | FDA | Application number: ANDA215506 (14)  Pyrimethamine is excreted in human milk. Because of the potential for serious adverse reactions in nursing infants from pyrimethamine and from concurrent use of a sulfonamide with pyrimethamine for treatment of some patients with toxoplasmosis, a decision should be made whether to discontinue nursing or to discontinue the drug, taking into account the importance of the drug to the mother. |
|  | BNFC | Significant amount is transferred in milk - avoid administration of other folate antagonists to infants.  Avoid breastfeeding during toxoplasmosis treatment (10). |
|  | WHO | Compatible with breastfeeding. Avoid if possible if the infant is premature or less than 1 month old. Monitor for side effects (hemolysis and jaundice). Avoid in G6PD-deficient infants (12). Recommended for lactating women as part of an ACT in combination with artesunate (11). |
|  | CDC | No specific information (13). |
| Piperaquine | EMA | Animal data suggest the excretion of piperaquine into breast milk, but no data are available on humans. Women taking Eurartesim should not breastfeed during their treatment (15). |
|  | BNFC | (with Artenimol) Manufacturer advises to avoid during breastfeeding as it is present in milk suggested by animal studies (10). |
|  | WHO | Recommended for lactating women as part of an ACT in combination with dihydroartemisinin (11). |
|  | CDC | No specific information (13). |

**Table S3.** Continued

| **Drug** | **Organization** | **Labelling** |
| --- | --- | --- |
| Mefloquine | FDA | Application number: ANDA076523, updated 2021 (16)  Excretion into breast milk appears to be minimal. Mefloquine is excreted in human milk in small amounts, the activity of which is unknown. Based on a study in a few subjects, low concentrations (3% to 4%) of mefloquine were excreted in human milk following a dose equivalent to 250 mg of the free base. Caution should be exercised when administered to a nursing woman. |
|  | BNFC | Present in milk but the risk to infants is minimal (10). |
|  | WHO | Recommended for lactating women as part of an ACT in combination with artesunate (11). |
|  | CDC | Mefloquine may be prescribed safely to infants, it is also safe for infants to be exposed to the small amounts excreted in breast milk. Pediatric dose: ≤9 kg: 4.6 mg/kg base (5 mg/kg salt) orally, once/week (10). |
| Primaquine | FDA | Application number: ANDA206043, updated 1/2022 (17)  It is not known whether primaquine is excreted in human milk. Because many drugs are excreted in human milk and because of the potential for serious adverse reactions in nursing infants from primaquine, a decision should be made whether to discontinue nursing or discontinue the drug, taking into account the importance of the drug to the mother. |
|  | BNFC | No information available; theoretical risk of hemolysis in G6PD-deficient infants (10). |
|  | WHO | Compatible with breastfeeding after 6 months post-partum in G6PD-normal subjects (11). |
|  | CDC | No information is available on the amount of primaquine that enters human breast milk. Primaquine can be used during breastfeeding if the infant is found to also have normal G6PD activity. Pediatric dose: 0.5 mg/kg base (0.8 mg/kg salt) (13). |

FDA - U.S. Food and Drug Administration; BNFC - British National Formulary for Children; WHO - World Health Organisation; CDC Centers for Disease Control and Prevention; ACT - artemisinin-based combination therapy; G6PD - glucose-6-phosphate dehydrogenase; mg - milligram; kg - kilogram

# Piperaquine Model Refinement

The published model was developed using data specifically from the Eurartesim formulation of piperaquine. When using this model to recover the PK across different formulations in clinical studies run in various sites (including the clinical lactation study (6)), an underprediction of the terminal half-life was noted. The model was, therefore, refined to ensure adequate recovery of the drug concentrations in plasma before milk concentrations were predicted. Interestingly, the new model was able to recover a range of clinical data but not those from the original Eurartesim dataset used. This highlights the need to check model performance for the study of interest before predicting milk concentrations.

Predicted plasma concentration profiles for the clinical lactation studies are shown in Figure 1 of the manuscript. Predicted profiles of an additional 3 studies (18–20) are shown below. In each case, 20 virtual trials of study n number were simulated. Demographics and dosing regimens were matched to those in the clinical study.

Simulations of the 2012 study by Hoglund et al. (18) were run as a 3-day course of 11 mg/kg piperaquine base daily at 0, 24, and 48h to a total of 500 virtual lactating subjects aged 18 - 45 years in 20 virtual trials (25 subjects in each trial).

Simulations of the 2021 study by Rijken et al. (19) were run as a 3-day course of 10 mg/kg piperaquine base daily at 0, 24, and 48h to a total of 460 virtual lactating subjects aged 18 - 45 years and allocated equally into 20 virtual trials (23 subjects in each trial).

Simulations of the 2012 study by Tarning et al. (20) were run as a 3-day course of 10 mg/kg piperaquine base daily at 0, 24, and 48h to a total of 480 virtual lactating subjects aged 18 - 45 years and allocated equally into 20 virtual trials (24 subjects in each trial).

The refined model was able to recover the observed concentration-time profiles (Figure 1 in the main manuscript and Figure S1).


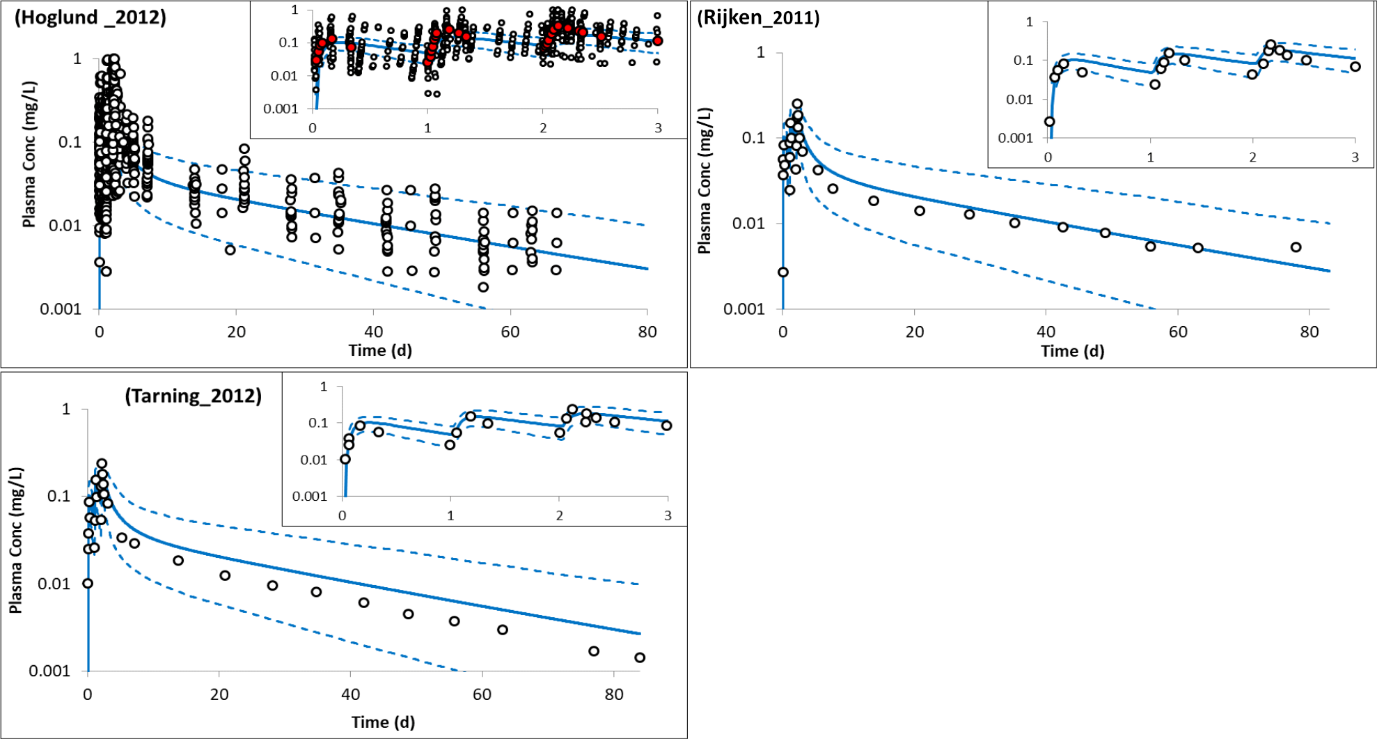


# Figure S1. Predicted (lines) vs. observed (circles) piperaquine plasma concentrations after maternal oral administration according to the trial designs described above. Solid lines: predicted means; Dashed lines: 5^th^ and 95^th^ percentiles.

# Lactation Model Code

***Files Provided***

The Lua scripts for the two published QSAR models are provided within the installation package for Version 22 and subsequent versions. For Version 21 (used in this work) the Lua script files are shared as part of this supplementary material.

Model 1 – Phase distribution model - Fleishaker, 1987, Atkinson & Begg, 1990 (21,22). There is one file for both acidic and basic compounds.

Lua script file name: Lactation-phase-distribution-model-acid-base-neutral

Model 2 – Log-transformed distribution model – Atkinson & Begg, 1988, Atkinson & Begg, 1990 (22,23). There are two files, one for acidic compounds and one for basic compounds.

Lua script file names:

Lactation-log-transformed-distribution-model-base

Lactation-log-transformed-distribution-model-acid

***Lua scripts use***

To use these scripts, users will first need to add the compound LogD values at a given milk pH.

To use the lactation model scripts in Simcyp Human Simulator V21:

- Open Simcyp Human Simulator V21 and navigate to the Compound tab.
- Go to Transport (Permeability Ltd Organ) and then the Lactation tab.
- Activate the lactation model via ‘Use lactation model’ option and select the ‘Perfusion-limited model’.
- Select ‘predicted’ and the ‘User Defined’ Lua option.
- Click on the ‘User Defined’ option to open the Lua script window.
-
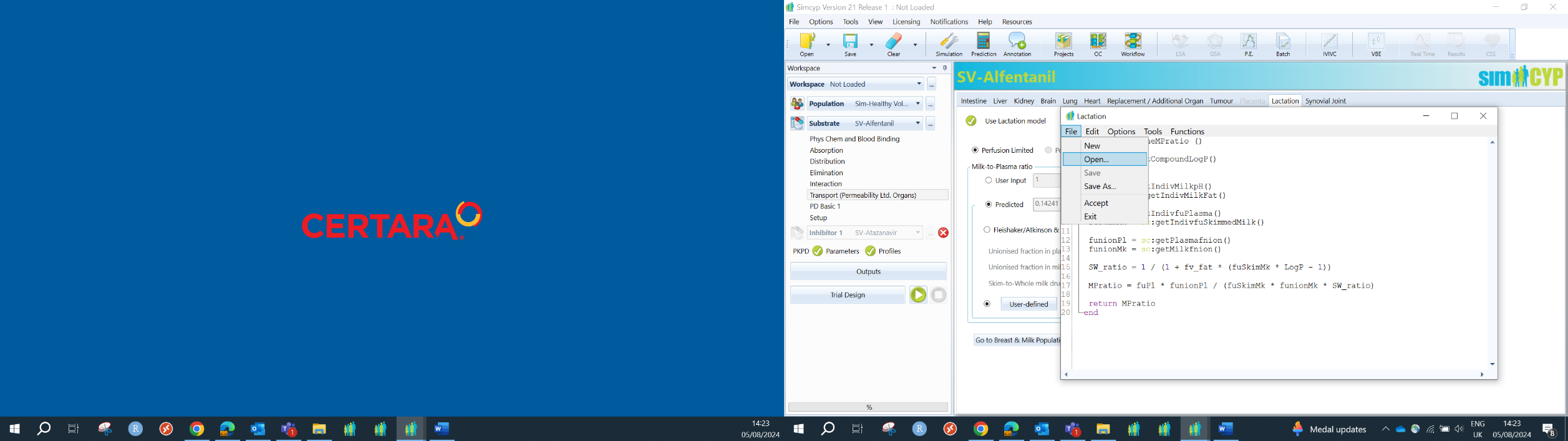
In the Lua script menu bar, go to ‘File’, select ‘Open’ and navigate the script file to open the Lua script (Figure S2).

# Figure S2. Screenshot of Lua script selection

- Provide the LogD value for your compound as instructed (Figure S3).
- Go to the ‘File; and select ‘Accept’ to apply the Lua script.

#
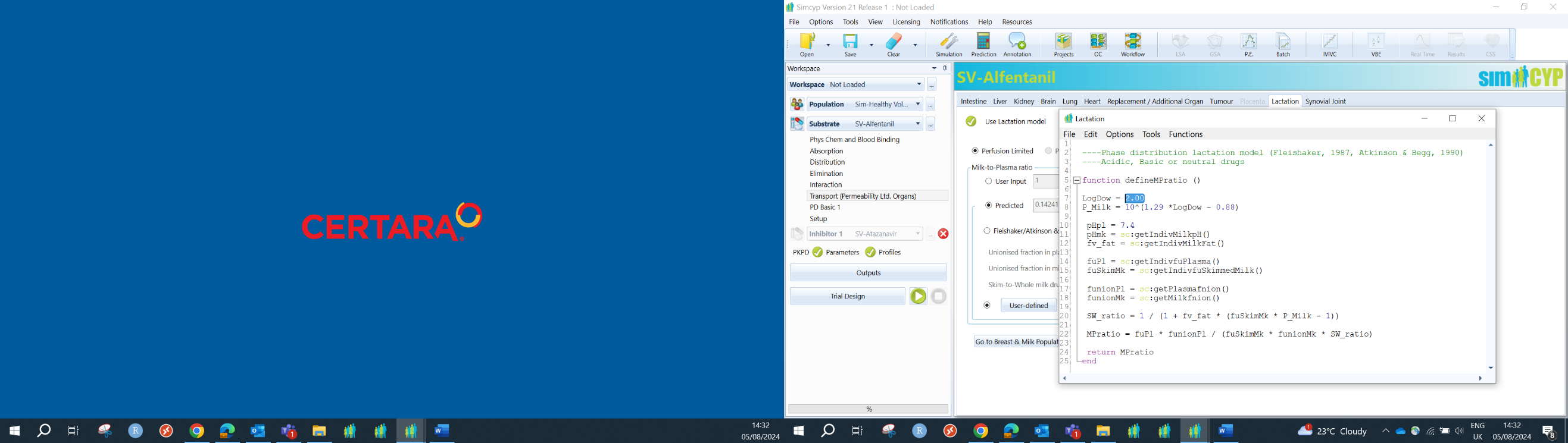
Figure S3. An example screenshot showing the provided logD of 2.00 in the script

Note: The Lua scripts utilize the milk parameters (i.e., milk pH and fat%) specified in the Population > Breast & Milk tab. Please ensure these parameters are correctly set for your simulation.

***Calculation of LogD at milk pH from LogD_7.4_/LogP***

If the value is not available, LogD can be calculated from the LogD_7.4_ or LogP value using the Simcyp prediction tool as follows:

- Open Simcyp and navigate to the menu bar.
- Go to Prediction Tool Box and select the LogP/LogD tab.
- Enter the physchem, LogP value and pH to calculate the LogD value for your compound.
- Note the predicted LogD value to add into your lactation model script.

# References

1. Law I, Ilett KF, Hackett LP, Page-Sharp M, Baiwog F, Gomorrai S, et al. Transfer of chloroquine and desethylchloroquine across the placenta and into milk in Melanesian mothers. Br J Clin Pharmacol. 2008 May;65(5):674–9.

2. Edstein MD, Veenendaal JR, Newman K, Hyslop R. Excretion of chloroquine, dapsone and pyrimethamine in human milk. Br J Clin Pharmacol. 1986 Dec;22(6):733–5.

3. Akintonwa A, Gbajumo SA, Mabadeje AF. Placental and milk transfer of chloroquine in humans. Ther Drug Monit. 1988;10(2):147–9.

4. Ogunbona FA, Onyeji CO, Bolaji OO, Torimiro SE. Excretion of chloroquine and desethylchloroquine in human milk. Br J Clin Pharmacol. 1987 Apr;23(4):473–6.

5. Ette EI, Essien EE, Ogonor JI, Brown-Awala EA. Chloroquine in human milk. J Clin Pharmacol. 1987 Jul;27(7):499–502.

6. Moore BR, Salman S, Benjamin J, Page-Sharp M, Yadi G, Batty KT, et al. Pharmacokinetics of Piperaquine Transfer into the Breast Milk of Melanesian Mothers. Antimicrob Agents Chemother. 2015 Jul;59(7):4272–8.

7. Edstein MD, Veenendaal JR, Hyslop R. Excretion of mefloquine in human breast milk. Chemotherapy. 1988;34(3):165–9.

8. Gilder ME, Hanpithakphong W, Hoglund RM, Tarning J, Win HH, Hilda N, et al. Primaquine Pharmacokinetics in Lactating Women and Breastfed Infant Exposures. Clin Infect Dis. 2018 Sep 14;67(7):1000–7.

9. Amneal Pharmaceuticals of New York LLC. Chloroquine Phosphate Tablet. U.S Food and Drug Administration. [Internet]. 2022 [cited 2024 May 27]. Available from: https://www.accessdata.fda.gov/drugsatfda_docs/label/2009/083082s050lbl.pdf

10. Paediatric Formulary Committee. British National Formulary for Children. BNF for Children (online) London: BMJ Group, Pharmaceutical Press, and RCPCH Publication. [Internet]. 2022. Available from: https://bnfc.nice.org.uk/

11. WHO guidelines for malaria, 16 October 2023. Geneva: WHO Global Malaria Programme;

12. Department of Child and Adolescent Health and Development. Breastfeeding and Maternal Medication Recommendations for Drugs in the Eleventh WHO Model List of Essential Drugs. Geneva: WHO, UNICEF; 2003.

13. Tan KR, Abanyie F. Section 5: Travel-Associated Infections & Diseases - Parasitic. In: CDC Yellow Book 2024 [Internet]. 2023 [cited 2024 May 27]. Available from: https://wwwnc.cdc.gov/travel/yellowbook/2024/infections-diseases/malaria

14. Teva Pharmaceuticals, Inc. Pyrimethamine Tablets. U.S Food and Drug Administration. [Internet]. 2021 [cited 2024 May 27]. Available from: https://www.accessdata.fda.gov/spl/data/90ea4054-7c0a-4971-be57-d72e482491b8/90ea4054-7c0a-4971-be57-d72e482491b8.xml

15. Alfasigma S.p.A. Eurartesim 160 mg/20 mg film-coated tablets. European Medicines Agency. [Internet]. 2016 [cited 2024 May 27]. Available from: https://www.ema.europa.eu/en/documents/product-information/eurartesim-epar-product-information_en.pdf

16. Hikma Pharmaceuticals USA Inc. Mefloquine Hydrochloride Tablets. U.S Food and Drug Administration. [Internet]. 2021 [cited 2024 May 27]. Available from: https://nctr-crs.fda.gov/fdalabel/services/spl/set-ids/43fde257-36ee-49ea-a03c-01a1a4e1da3d/spl-doc?hl=mefloquine%20hydrochloride#_Refi4i_nursing_mothers_id_33d7ace9-997c

17. AvKARE. Primaquine Phosphate Tablets. U.S Food and Drug Administration. [Internet]. 2022 [cited 2024 May 27]. Available from: https://www.accessdata.fda.gov/spl/data/d57d3921-0857-6640-e053-2a95a90a6116/d57d3921-0857-6640-e053-2a95a90a6116.xml

18. Hoglund RM, Adam I, Hanpithakpong W, Ashton M, Lindegardh N, Day NP, et al. A population pharmacokinetic model of piperaquine in pregnant and non-pregnant women with uncomplicated Plasmodium falciparum malaria in Sudan. Malar J. 2012 Dec;11(1):398.

19. Rijken MJ, McGready R, Phyo AP, Lindegardh N, Tarning J, Laochan N, et al. Pharmacokinetics of dihydroartemisinin and piperaquine in pregnant and nonpregnant women with uncomplicated falciparum malaria. Antimicrob Agents Chemother. 2011 Dec;55(12):5500–6.

20. Tarning J, Rijken MJ, McGready R, Phyo AP, Hanpithakpong W, Day NPJ, et al. Population Pharmacokinetics of Dihydroartemisinin and Piperaquine in Pregnant and Nonpregnant Women with Uncomplicated Malaria. Antimicrob Agents Chemother. 2012 Apr;56(4):1997–2007.

21. Fleishaker JC, Desai N, McNamara PJ. Factors affecting the milk-to-plasma drug concentration ratio in lactating women: physical interactions with protein and fat. J Pharm Sci. 1987 Mar;76(3):189–93.

22. Atkinson HC, Begg EJ. Prediction of drug distribution into human milk from physicochemical characteristics. Clin Pharmacokinet. 1990 Feb;18(2):151–67.

23. Atkinson H, Begg E. Prediction of drug concentrations in human skim milk from plasma protein binding and acid‐base characteristics. Brit J Clinical Pharma. 1988 Apr;25(4):495–503.
